# First Complete Genome Sequence of *Staphylococcus xylosus*, a Meat Starter Culture and a Host to Propagate *Staphylococcus aureus* Phages

**DOI:** 10.1128/genomeA.00671-14

**Published:** 2014-07-10

**Authors:** Simon J. Labrie, Lynn El Haddad, Denise M. Tremblay, Pier-Luc Plante, Jessica Wasserscheid, Jeannot Dumaresq, Ken Dewar, Jacques Corbeil, Sylvain Moineau

**Affiliations:** a Département de Biochimie, de Microbiologie et de Bio-informatique, Faculté des Sciences et de Génie, Groupe de Recherche en Écologie Buccale, Faculté de Médecine Dentaire, Félix d’Hérelle Reference Center for Bacterial Viruses, Université Laval, Québec, Canada; b Département de Médecine Moléculaire, Faculté de Médecine, Université Laval, Québec, Canada; c Department of Human Genetics, McGill University and Genome Quebec Innovation Centre, Montréal, Canada; d Département de Microbiologie et d’Infectiologie, Centre Hospitalier Affilié Universitaire Hôtel-Dieu de Lévis, Lévis, Québec, Canada

## Abstract

*Staphylococcus xylosus* is a bacterial species used in meat fermentation and a commensal microorganism found on animals. We present the first complete circular genome from this species. The genome is composed of 2,757,557 bp, with a G+C content of 32.9%, and contains 2,514 genes and 79 structural RNAs.

## GENOME ANNOUNCEMENT

*Staphylococcus xylosus* is a coagulase-negative species found on the skin and mucosa of animals (1). It is also used as a starter culture for meat fermentation (2). This species is generally recognized as safe for humans, although some strains have been associated with animal opportunistic infections (3–5).

Strain SMQ-121 is used in fermented sausage processes and is being considered a production host for polyvalent myophages to control unwanted *Staphylococcus aureus* cells (13). The species of SMQ-121 was confirmed through 16S analysis and an API Staph kit (13). We sequenced its genome to confirm its safety by the absence of genes coding for known virulence factors.

The genome was sequenced with Illumina and Pacific Biosciences platforms. For Illumina, genomic DNA was extracted (6) and libraries were prepared with the Nextera XT DNA sample preparation kit and Nextera mate-pair sample preparation kit according to instructions to obtain paired-end and mate-pair reads. Libraries were sequenced using a MiSeq reagent kit (2 × 151 nucleotides [nt] and 2 × 250 nt for paired-end and mate-pair libraries, respectively) on a MiSeq. We also used ultrapure large size chromosomal DNA fragments for PacBio sequencing to close the genome. The DNA was extracted using Genomic-tip 20/G (Qiagen) according to the manufacturer’s instructions, except that 10 times more cells were used. Genomic DNA was sheared to 20 kb using g-tubes (Covaris) and used to prepare a Pacific Biosciences RS library. A single SMRT cell was sequenced to generate a data set including 25,016 unique subreads of minimum length 3 kb (average length, 7.314 kb). The genome was assembled using a combination of HGAP2/Celera/Quiver following Pacific Biosciences recommendations. Quiver was rerun until all base calls were stable. Base calling accuracy was additionally verified using Illumina reads that were aligned with Blat (7). With the exception of a single 50-bp interval of high AT content, where Illumina coverage was reduced, the assembly was concordant with perfect and full-length aligning Illumina reads. The genome of 2,757,557 bp has an average G+C content of 32.9%. The circular genome was annotated using RAST (9), and it contains 2,514 genes and 79 structural RNAs.

Coding sequences were analyzed to detect toxin genes by using VirulenceFinder (http://cge.cbs.dtu.dk/services/VirulenceFinder/) and by comparing the protein sequences using BLASTP (12) with sequences in virulence factor databases (VFDB [10] and DBETH [8]). We have not detected genes coding for toxins or virulence factors. Proteins were also compared with an antibiotic resistance gene database (ARG-ANNOT [11]), and we found four resistance genes. Two genes encode proteins that belong to the major facilitator superfamily (PF07690 and cd06174) involved in phenicol and fluoroquinolone resistance. Another gene encodes a putative aminoglycoside 3′-phosphotransferase for resistance to aminoglycosides, and the last one encodes trimethoprim resistance. Although the deduced proteins share more than 70% identity with known antibiotic resistance proteins, *S. xylosus* SMQ-121 was found to be sensitive to amikacin, chloramphenicol, ciprofloxacin, and trimethoprim.

### Nucleotide sequence accession number.

The annotated sequence of *S. xylosus* SMQ-121 was deposited under GenBank accession number CP008724.
